# Urgent need to develop evidence-based self-help interventions for mental health of healthcare workers in COVID-19 pandemic

**DOI:** 10.1017/S0033291720001385

**Published:** 2020-04-28

**Authors:** Lei Yang, Juan Yin, Duolao Wang, Atif Rahman, Xiaomei Li

**Affiliations:** 1 School of Nursing, Health Science Center, Xi'an Jiaotong University, Xi'an, Shaanxi, PR China; 2 Liverpool School of Tropical Medicine, Liverpool, UK; 3 Department of Psychological Sciences, University of Liverpool, Liverpool, UK

The pandemic of COVID-19 has rapidly spread to over 200 countries and territories in the past 4 months. To save lives and minimize transmission, millions of healthcare workers are working in front lines worldwide by putting themselves at high risk from the disease. All of them are working under extreme pressure and experiencing great psychological distress in such a challenging situation. In addition to the huge psychological pressure, healthcare workers around the world face morale and burnout issues as they have to make impossible decisions (Greenberg, Docherty, Gnanapragasam, & Wessely, 2020). It is encouraging that the World Health Organization (WHO) and many institutions have proposed guidelines to provide psychological assistance for healthcare workers during this pandemic.

However, it is important to ensure that evidence-based intervention strategies are employed so that already over-stretched resources can be maximized. Many barriers limit the implementation of conventional evidence-based interventions in this emergent setting. First, traditional face-to-face psychotherapy is hard to implement immediately because of the quarantine policy for minimizing transmission of the virus. Second, not all healthcare workers willingly participate in the group or individual psychological interventions, as evidenced by recent experiences from China (Chen et al., 2020). Third, current evidence-based interventions commonly target single mental disorders, whereas a range of psychological responses and mental disorders are experienced by populations facing an emerging epidemic outbreak. Fourth, COVID-19 has spread worldwide including many low- and middle-income countries (LMICs) where significant gaps exist in access to mental health services, but traditional evidence-based interventions generally require substantial mental health resources.

Given these challenges, urgent attention needs to be given to researching strategies to improve access to evidence-based psychological interventions for frontline workers, especially in LMICs. Self-help interventions offer a promising avenue because such interventions can be delivered through a variety of media, and self-help has been shown to be effective for a range of mental health problems (Scott, Webb, & Rowse, 2015). The WHO have developed an evidence-based self-help intervention called Self-Help Plus (SH+) for managing stress and coping with range of adversities (Epping-Jordan et al., 2016). SH+ is based on principles of Acceptance Commitment Therapy and adopts a guided self-help format comprising pre-recorded audio sessions and an illustrated self-help book. Evidence has shown that guided SH+ can be rapidly implemented and achieved meaningful improvement in psychological distress, posttraumatic stress disorders (PTSD) and depression symptoms among south Sudanese female refugees (Tol et al., 2020). SH+ is easily adaptable to different cultures and languages and both meaningful and safe for people with and without mental disorders. We are working to adapt a Chinese version of SH+ for frontline healthcare workers, and expect it can provide a useful reference for many countries to better respond to safeguarding the mental health of their frontline workers.

In summary, we see a grave need for the development of evidence-based self-help interventions for frontline healthcare workers to protect their mental health during the COVID-19 pandemic. They are the true heroes sacrificing a lot to save our lives today, it is our responsibility to ensure their current and future wellbeing.

## References

[ref1] Chen, Q., Liang, M., Li, Y., Guo, J., Fei, D., Wang, L., … Zhang, Z. (2020). Mental health care for medical staff in China during the COVID-19 outbreak. The Lancet. Psychiatry, 7(4), e15–e16. doi: 10.1016/s2215-0366(20)30078-x. 32085839 PMC7129426

[ref2] Epping-Jordan, J. E., Harris, R., Brown, F. L., Carswell, K., Foley, C., Garcia-Moreno, C., … van Ommeren, M. (2016). Self-Help Plus (SH + ): A new WHO stress management package. World Psychiatry, 15(3), 295–296. doi: 10.1002/wps.20355. 27717271 PMC5032500

[ref3] Greenberg, N., Docherty, M., Gnanapragasam, S., & Wessely, S. (2020). Managing mental health challenges faced by healthcare workers during COVID-19 pandemic. BMJ, 368, m1211. doi: 10.1136/bmj.m1211. 32217624

[ref4] Scott, A. J., Webb, T. L., & Rowse, G. (2015). Self-help interventions for psychosis: A meta-analysis. Clinical Psychology Review, 39, 96–112. doi: 10.1016/j.cpr.2015.05.002. 26046501

[ref5] Tol, W. A., Leku, M. R., Lakin, D. P., Carswell, K., Augustinavicius, J., Adaku, A., … van Ommeren, M. (2020). Guided self-help to reduce psychological distress in South Sudanese female refugees in Uganda: A cluster randomised trial. The Lancet. Global Health, 8(2), e254–e263. doi: 10.1016/s2214-109x(19)30504-2. 31981556 PMC9899129

